# Unstable pelvic fractures in women: implications on obstetric outcome

**DOI:** 10.1007/s00264-023-05979-4

**Published:** 2023-09-15

**Authors:** Amit Davidson, Vasileios P. Giannoudis, Georgios Kotsarinis, Emmanuele Santolini, Constantinos Tingerides, Anish Koneru, Nikolaos K. Kanakaris, Peter V. Giannoudis

**Affiliations:** 1 Academic Department of Trauma and Orthopaedics, School of Medicine, University of Leeds, Clarendon Wing, Floor D, Great George Street, Leeds General Infirmary, Leeds, LS1 3EX UK; 2 Department of Diagnostic and Interventional Radiology, Leeds General Infirmary, Leeds Teaching Hospitals NHS Trust, Leeds, UK; 3 NIHR Leeds Biomedical Research Center, Chapel Allerton Hospital, Leeds, UK

**Keywords:** Pelvic fracture, Delivery method, Obstetric concerns, Hardware removal, Radiographic measurements

## Abstract

**Purpose:**

Obstetric outcomes in women following pelvic injuries requiring surgical fixation is not thoroughly known. We aimed to evaluate if radiographic measurements (RMs) can be used to provide information on delivery methods outcome after these injuries, and to evaluate if metal work removal is required prior to delivery.

**Method:**

A retrospective study in a level 1 trauma centre of female patients with pelvic fractures treated operatively, aged 16–45 at the time of injury. Participants completed a questionnaire regarding their obstetric history. RM evaluating pelvic symmetry, displacement, and pelvimetry were conducted on postoperative radiographs and CT scans. Patients who gave birth after the injury were divided to two groups according to the delivery method: vaginal delivery (VD) and caesarean section (CS). These two groups RM were compared.

**Results:**

Forty-four patients were included, comparison of the RM of patients who delivered by CS (9) and patients who had only VD (11) showed no significant difference between the groups. Two patients underwent a trial of VD who subsequently underwent urgent CS due to prolonged labour, their RM were below the average and their pelvimetry measurements were above the cut-off for CS recommendation. Eleven patients had uncomplicated VD, all had retained sacroiliac screws at the time of delivery and one patient had an anterior pubic plate.

**Conclusion:**

Postoperative RM did not show an effect on delivery method of women after pelvic fracture fixation. A relatively high number of patients who underwent normal vaginal delivery had retained sacroiliac screws. These findings can form the foundation for larger cohort studies.

**Supplementary Information:**

The online version contains supplementary material available at 10.1007/s00264-023-05979-4.

## Introduction

Pelvic fractures sustained at young ages can be complex injuries associated with marked morbidity [1, 2]. Moreover, young females who are of childbearing age with pelvic fractures can have subsequent obstetric concerns [3, 4].

Normally, the physiological process of pregnancy and labour is associated with pelvis widening and reshaping to accommodate the foetus [5, 6]. For women who sustained a severe pelvic injury, this normal pelvic expansion and movement can be altered. The degree of displacement and asymmetry of the pelvis and the retained hardware could theoretically be an obstacle to normal vaginal delivery by reducing the normal pelvic birth canal diameter.

There is a paucity in literature investigating obstetric outcomes of women post pelvic fractures. Furthermore, many of the patients presented in previous studies are those who were treated non-operatively [7–10]. Reported caesarean section (CS) rates after pelvic fractures are higher than the population norm [9]; this includes a large registry from Scandinavia which was recently published [11]. The reason for the high rate of CS is unknown. It can be assumed that the lack of literature regarding the safety of vaginal delivery after pelvic injury may influence patients and obstetricians to prefer an elective CS.

In obstetrics, pelvimetry is routinely used to predict and assess feasibility for vaginal delivery [12]. There are acceptable pelvimetry radiographic measurements ranges that are used for recommendation of caesarean section delivery method [13]. To date, these measurements were not recorded as a quantitative tool to assist patients and care givers in the decision of delivery method after severe pelvic fractures. In addition to pelvimetry, there are orthopaedic radiographic measurements that evaluate the degree of displacement after pelvic injury and surgery which provide radiographic quantitative measurements [14].

The primary objective of this study was to evaluate the obstetric outcomes in women of childbearing age following severe pelvic injury who underwent surgical fixation. Secondary aims were to investigate if radiographic measurements can be used to provide information for recommendation of delivery method after these injuries, and if metal work requires removal prior to delivery.

## Patients and methods

Following acquisition of Institutional Review Board approval, data regarding young women of childbearing age (16–45 at time of injury) with severe pelvic ring injuries, treated at a single level 1 trauma centre between the years 2009 and 2019, were obtained. Inclusion criteria were female patients diagnosed with pelvic fracture who were treated operatively and had comprehensive postoperative obstetric history records (patient questionnaires and electronic data reports).

Operatively treated females were identified retrospectively through hospital electronic records. Participants were contacted by mail and emails to complete a questionnaire to evaluate data regarding obstetric related concerns, pregnancy rates and delivery methods (Appendix 1). This questionnaire was based on previously designed questionnaires in similar studies [7, 10]. In addition, obstetric data concerning birth rate, delivery methods and pregnancy or delivery-related complications were collected by the electronic regional medical database.

During the pre-specified study period, eighty-one female patients were treated operatively for an acute pelvic injury. However, comprehensive postoperative obstetric history (patient questionnaires and electronic data reports) was available for 44 patients. This patient cohort group formed the basis of the study. Table 1 summarises data regarding demographics, associated injuries, mechanism of injury and fixation modality for these patients, which were obtained from the hospital electronic data base.

**Table 1:** Demographics, associated injuries, mechanism of injury, fracture classification and fixation modality of patients included in the study

Number of patients	44
Age [years] (mean, range)	25 (16–42)
Fracture classification	
LC 1	21
LC 2	4
LC 3	11
APC 2	3
APC 3	3
VS	2
Mechanism of injury	
Motor vehicle collision	25
Pedestrian versus car	6
Fall from height	8
Other	5
Associated injures	
ISS score, mean (range, SD)	15 (34–9, 7.2)
Associated injures, no. of patients	
Head injury	9
Lower extremity	10
Upper extremity	7
Chest	4
Abdominal	8
Bladder	1
Spine	10
Fracture fixation modality	
Posterior fixation	
Sacroiliac screw	39
Plate	1
Anterior fixation	
External fixation	24
Retropubic screw	7
Plate	7
Hardware removal	4
Time from initial operation to hardware removal [months] (mean, range)	20 (11–41)

Pelvic fracture classification was done by two of the authors, using the institutional Picture Archiving and Communication System (PACS); patients’ radiographs and computed tomography (CT) scan upon arrival were analysed. Fractures were classified according to Young Burgess and AO/OTA classifications [15]. When inconsistency between the assessors was found, the radiographs were re-evaluated until a consensus regarding the classification was reached.

**Fig. 1:**
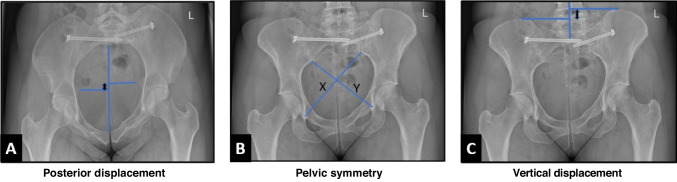
Illustration demonstrating the postoperative displacement measurements measured on postoperative radiographs in different views. **A** This demonstrates displacement evaluation in the anterior posterior (AP) direction. On postoperative inlet plain films, a straight line connecting anterior midline to posterior midline was drawn. A line perpendicular to this midline line was drawn out to each ischial spine. The distance between these two lines, represented by a two headed arrow, is taken as the AP displacement. **B** This demonstrates evaluation of pelvic symmetry which was performed on AP radiographs according to method described by Lefaivre et al. [17]. On an AP radiographs, a line is drawn from the inferior aspect of the sacroiliac joint to the contralateral teardrop inferior aspect. The length of the two lines is represented by the letters X and Y. The longer line (X) is subtracted from the second line (Y), yielding a pelvic asymmetry value (X-Y). **C** This demonstrates the vertical displacement measurements we applied technique which was described by Henderson et al. [16]. A straight midline was drawn through the lower lumber and upper sacral area. Horizontal lines perpendicular to this center line is drawn across the superior most point of each iliac wing. The distance between these two horizontal lines, illustrated by a two-headed arrow, is taken as the posterior displacement

**Fig. 2:**
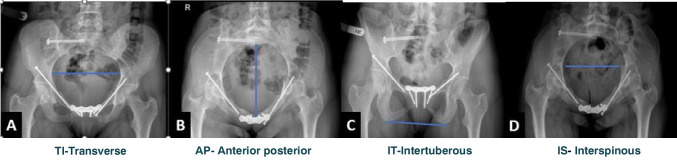
Illustration demonstrating the radiographic pelvimetry measurements. These measurements were obtained based on the Colcher-Sussman technique [13] on postoperative radiographs in different views. **A** The pelvic transverse inlet diameter (TID); on an anteroposterior pelvic view, a line is drawn from the iliopectineal line which forms the widest diameter of the pelvic inlet. **B** The anterior posterior inlet distance measurement (APD) was measured by a line drawn from the anterior aspect of the pubic symphysis to the anterior aspect of the sacrum in the pelvic inlet view. This measurement is a modification to the measurement described by Colcher-Sussman technique which was described as measurement which was obtained on lateral projections. Despite the difference in the projection, the same reference points were applied. **C** The pelvic outlet transverse distance defined as intertuberous distance (ITD) was obtained on a pelvic outlet view. A line was drawn from the most inferior part of the ischial tuberosity of each side. **D** The mid pelvic transverse diameter defined as Interspinous distance (ISD) was measured on pelvic inlet view. A line connecting the tip of the ischial tuberosity in each side was drawn

Fixation quality was evaluated by postoperative radiographs anteroposterior (AP), inlet and outlet pelvic views. These radiographic measurements were conducted on the most recent radiographs available with a minimal time of 12 months after the injury. The measurements were calibrated using the screw size inner diameter calibration methods [16]. Vertical displacement was measured as described by Henderson et al. [17]. Pelvic symmetry was evaluated on AP radiographs according to Lefaivre et al. [18], and illustrations of these measurements are shown in Fig. 1. Pelvimetry measurements were performed on the same postoperative radiographs according to Colcher-Sussman technique [19]. Description of the pelvimetry measurements and illustrations are shown in Fig. 2. For the pelvimetry measurements described by Colcher-Sussman on the lateral projection, modified measurements using post operative inlet radiographs were obtained, as lateral radiographs are not routinely used in our daily practice.

**Fig. 3:**
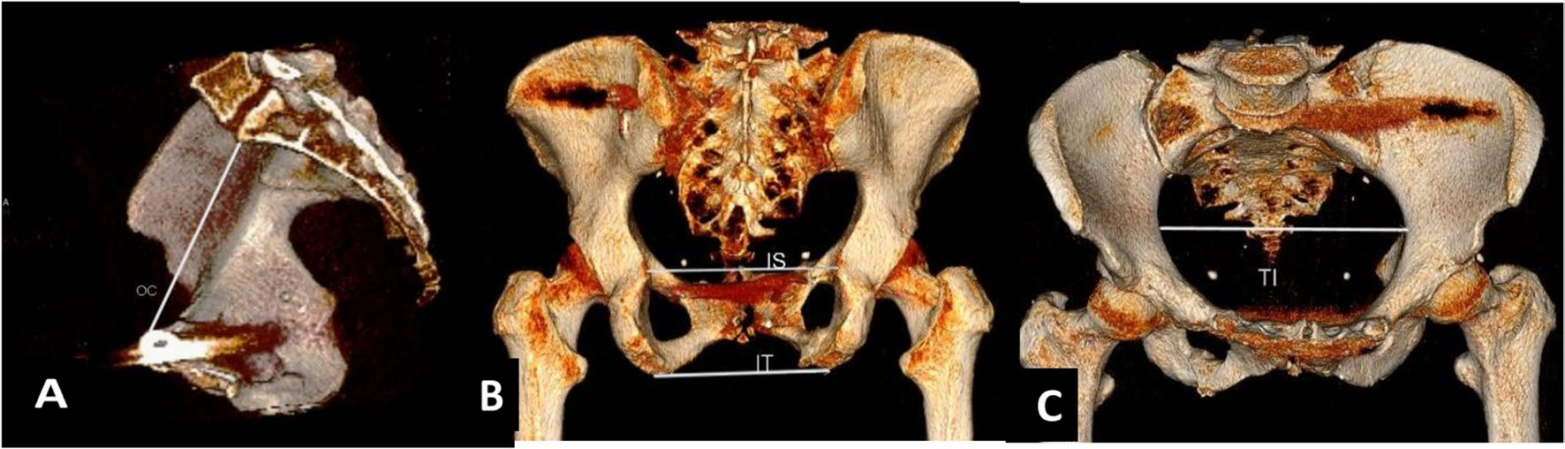
3D volume rendered reconstructions of the bony pelvis are given in **A** lateral, **B** posterior and **C** anterior cranial views after ‘cutting’ the pelvis in different planes. White lines indicate the 2D measurement dimensions, as described by Kaufmann et al. [20]. **A** Lateral view after cutting the pelvis in a (para)median plane, measurement of obstetric conjugate (OC) also defined as anterior posterior distance (APD). This measurement is the shortest distance from promontory to the superior aspect of the symphysis. **B** A posterior view reconstruction, interspinous (IS) distance measured as the distance between the spinous processes in this view, and intertuberous (IT) distance measured as the distance between tuberous processes. **C** Lateral view reconstruction, transverse inlet (TI) measured as the widest transverse pelvis brim distance

In case a CT scan was performed 12 months or more after the initial injury, CT-based pelvimetry measurements were obtained rather than plain radiographs, due to superior reproducibility and accuracy shown in CT-based measurements [20]. Postoperative scans were performed on multidetector computed tomography (MDCT) scanners using helical acquisition and reconstructed in 1 mm slices. Three-dimensional (3D) volume rendering was performed using IMPAX Volume Viewing software, 4.0; Clinapps 7.0.282.0; Agfa Healthcare, Belgium. The CT measurements were based on the technique described by Kaufmann et al. [21]. Measurements were obtained on volume rendered images (3D reconstruction). Similar to the plain radiographs, 4 measurements were obtained. Description of these measurements and illustrations are shown in Fig. 3.

Patients who gave birth after the injury were divided to two groups according to the delivery method: vaginal delivery (VD) group and caesarean section (CS) group. Reduction quality measurements and pelvimetry measurements were compared for these two groups. The general pelvimetry cut-off measurements used in this study as recommendation for CS include TID < 115, ISD < 90, APD < 115, APD + TID < 220 [13].

Data was analysed using SPSS software. Categorial adjustment variables were analysed by chi-squared test. Continuous variables were analysed through Pearson and ANOVA tests. Statistical significance was set at a *p* value of ≤0.05. For the sample size power analysis two-sided confidence interval of 95% (1-α) and power (1-β) of 80% fora similar group characteristics was applied.

## Results

We found twenty-one patients who had full records of post injury pregnancies sustaining severe pelvic injury which were treated surgically. Eleven patients delivered by VD and 9 had CS; one patient had an early spontaneous abortion (week 11) and did not have additional pregnancies. Postoperative obstetrics data including number of pregnancies, delivery method, abortions and failed vaginal delivery attempts are presented in Table 2. Two patients underwent a trial of VD who subsequently required urgent CS due to prolonged labour. All other CS deliveries were planned following joint decision making between the treating obstetrician and patient. Regarding retained hardware during delivery, three of the nine patients who had CS had retained anterior fixation retropubic screws, two patients had pubic plates, eight had sacroiliac screws and 1 had a posterior plate in situ. All the eleven patients in the VD group had retained sacroiliac screws at the time of delivery and one patient had anterior pubic plate. For the patients with failed vaginal delivery attempt, one patient had removal of hardware due to malposition of a screw before her delivery and the other had one sacroiliac screw retained.

**Table 2:** Postinjury obstetrics data

Obstetric data	
Number of patients who become pregnant	21
Number of pregnancies	27
Number of vaginal deliveries	16
Number of caesarean sections	7
Caesarean section after failed vaginal delivery attempt	2
Number of miscarriages	2

Comparison of the radiographic measurements, including pelvimetry and postoperative reduction measurements, of patients who delivered by CS (9) and patients who had only vaginal delivery (11) is presented in Table 3. No significant difference was found in the radiographic measurements of these two groups. The radiographic measurements of the two patients that delivered by emergency CS were unexceptional, the displacement radiographic measurements were below the cohort average and their pelvimetry measurements were above the cut-off for CS recommendation. Radiographic measurements including pelvimetry and postoperative reduction evaluation were obtained on postoperative radiographs with the exception of seven patients for whom a postoperative CT scan was available. Results of the hole study cohort (44 patients) radiographic measurements including mean, range and standard deviation for each variable are presented in Table 4.

**Table 3:** Radiographic measurements of patients who delivered by caesarean section (9) compared to measurements obtained for patients who only had vaginal delivery (11)

Radiographic measurement	Delivery	Mean	SD	*p* value
ITD	VD	11.6	.9	0.09
	CS	10.8	1.1	
TID	VD	12.0	.76	0.73
	CS	11.9	1.1	
ISD	VD	9.3	1.1	0.88
	CS	9.3	.9	
APD	VD	13.9	1.2	0.96
	CS	13.9	1.6	
APD+TID	VD	26.0	1.7	1.00
	CS	25.9	2.2	
Pelvic asymmetry	VD	8.3	6.13	0.97
	CS	8.2	7.74	
Coronal displacement	VD	4.8	2.75	0.37
	CS	6.5	4.53	
Vertical displacement	VD	4.2	3.38	0.98
	CS	4.2	2.26	

*VD* vaginal delivery, *CS* caesarean section, *ITD* intertuberous diameter, *TID* transverse inlet diameter, *ISD* interspinous diameter, *APD* anteroposterior distance

**Table 4:** Radiographic measurements for all patient cohort

Radiographic measurements, mm, mean (range, SD)	
Displacement measurements	
Pelvic asymmetry	6.9 (20–0,6.5)
Vertical displacement	3 (11–0, 2.8)
Coronal displacement	4.2 (14–0, 3.35)
Pelvimetry	
TID	11.8 (9.2–17, 1.3)
ITD	11.1 (8.3–15, 1.4)
APD	13.8 (11–17, 1.4)
ISD	9.3 (7.1–12, 1.2)

*TID* transverse inlet diameter, *ITD* intertuberous diameter, *APD* anteroposterior distance, *ISD* interspinous diameter

The most common stated reason by the patients for the CS delivery was a recommendation by the obstetrician (4 patients), whereas in three patients, it was their own preference; two patients had expressed other obstetric concerns not related to the pelvic injury as a reason for their CS. None of these patients had CS delivery before the injury. Fifty-three percent of the patients who completed the questionnaire stated that they were ‘afraid’ of becoming pregnant following their injury.

## Discussion

This is the first study that uses pelvimetry measurement to evaluate obstetric outcomes for women who sustained severe pelvic injury. Our goal was to provide the methodology how to obtain parameters to support recommendations for delivery method after these injuries. Our results did not show a clear correlation between delivery methods and the radiographic measurements, neither in pelvimetry nor in the postoperative displacement measurements. We report on a relative high number of patients who underwent uneventful VD after pelvic fixation with retained hardware.

Our study is limited by a small sample size, although the number of women with deliveries after pelvic surgical fixation we present is the highest published to date. Furthermore, information related to the injury and subsequent care was retrospectively collected, and the obstetric history was obtained by questionnaires and could have recall bias. We could not directly contact the treating obstetricians of patients in the cohort but used electronic hospital records to collect obstetric data in addition to data collected by the questionnaires. Despite these limitations, this study provides data regarding obstetrics outcome after severe pelvic injury and offers a methodology that could be used to obtain radiographic measurements for these patients.

Pelvic fractures in young age usually occur following high energy trauma and are associated with long-term morbidity on patient’s functional status and quality of life [22]. Surgical indications are based on fracture displacement, associated injuries and patient functional status. In young patients, restoration of pelvic symmetry and fracture reduction quality improved patients functional outcome [1]. Surgical indications for minimally displaced fractures are less clear [23, 24]. Surgical treatment should be considered carefully as these surgeries often have major complication [25]. In young woman, additional factors regarding future obstetrics concerns should be considered when making treatment decisions. The effect of asymmetrical pelvis on future pregnancies and the effect of retained hardware on pelvic expansion are not thoroughly known [26].

CS rates after pelvic injury and especially after pelvic fixation are higher than the population norm ranging from 9 to 88% [9]. In our study, we had 43% (9/21) of patients who delivered by CS which is higher than the local population norm which stands at 32.8% [27]. As previously mentioned, a recent study from a Scandinavian registry showed higher CS rate also ten years following the original injury [11]. We believe like previous reports [10, 11] that the high CS rates are related to the lack of consensus and published evidence which will support patients, obstetricians and orthopaedic surgeons in recommending a trial of vaginal delivery. The high percentage of patients (51%) who stated that they are afraid of becoming pregnant after their injury in our questionnaire reflects this point.

Using objective data to support delivery method recommendation can reduce unnecessary CS in women after pelvic injury. Pelvimetry methods have been used for decades for similar purposes [19]. We applied these measurements to our study cohort. Our results did not show a clear correlation between delivery methods and the radiographic measurements when dividing the cohort to patients who delivered by CS and VD. The radiographic measurements of two patients who failed VD attempt in our cohort were in the average range; the reason for the failed delivery attempt cannot be clearly associated to the pelvic displacement and asymmetry. On the contrary, we did show a trend for one of the pelvimetry measurements (ITD) to be lower in the CS group (*p* value 0.09). Our study cohort is relatively small, and therefore, we recommend further studies to be performed to evaluate the association between radiographic measurements and obstetric outcome.

The postoperative pelvic displacement and symmetry radiographic measurements are an additional subjective measurement tool that should be applied. They provide information regarding pelvic asymmetry which in theory can influence the obstetric outcome and address the unique characteristics of women who sustained severe pelvic injury. Previous study investigating obstetric outcomes after pelvic injury divided patients according to grades of fracture displacement (0–4, 4–10 and above 10 mm of displacement) [10]. Nonetheless, we believe that this method of measurement lacks the ability to assess symmetry and is inaccurate in case there is malalignment in multiple planes.

The influence of retained hardware of pelvic fixation on pregnancy progression and delivery is unknown. There is lack of data especially reporting on VD with retained hardware after pelvic fixation. Vallier et al. [10] reported six patients who had postinjury uneventful VD after surgical fixation; three of these patients had retained trans symphysial plating. Another study reported on four patients having VD after pelvic fixation, but it is unclear if hardware was retained at delivery time [7]. In our study, we report on 11 patients with 16 uneventful vaginal deliveries all with intact hardware. All 11 patients had retained sacroiliac screws; three had bilateral sacroiliac screws and one patient had retained trans symphysial plating. Trans symphyseal plating theoretically could influence progression of vaginal delivery as studies have shown that the pubis symphysis width increases during labour [28]. Despite these concerns, our finding supports previous recommendations that intact pelvic fixation hardware, anterior or posterior is not an indication for CS [9]. One of the patients that failed VD attempt in our cohort had a sacroiliac screw retained at delivery time, although the reason for the failed VD attempt cannot be directly related to retained hardware. Despite our findings, a larger study is needed in order to provide a more solid ground for recommending a trail of vaginal delivery for all cases.

In conclusion, postoperative radiographic measurements did not show an effect on delivery method of women after pelvic fracture fixation. A relatively high number of patients that underwent normal vaginal delivery with retained sacroiliac screws was found. We recommend further studies with a large number of patients to provide a more solid recommendation for these important concerns. We believe that the measurement method presented in this study is accessible and reproducible and can be used for further studies.

### Supplementary information

ESM 1 (DOCX 16 kb)

## Data Availability

Raw data were generated from electronical records of the participating medical centre. Derived data supporting the findings of this study are available from the corresponding author [A.D] on request.
