# Supplementary material for: Unstable pelvic fractures in women: implications on obstetric outcome
Source: Int Orthop. 2023 Sep 15;48(1):235–41. doi: 10.1007/s00264-023-05979-4 (PMC10766730; doi:10.1007/s00264-023-05979-4)
Supplement: Supplementary file 1 — (DOCX 16 kb) [file 264_2023_5979_MOESM1_ESM.docx]

**Name____________ NHS number_____________ Date of birth _______**

**Date of completion of the questioner _________**

**Questionnaire - pregnancy and delivery**

1. Were you ever pregnant *before* your pelvic injury? **YES NO**

**If yes,**

**Number of pregnancies _____**

what have been the outcomes of your pregnancies:

Vaginal delivery _______

Forceps/vacuum delivery _______

C-section _______

Miscarriage _______

Ectopic/Termination _______

**Please provide detailed information:**

1. Have you been pregnant *after* your pelvic injury? **YES NO**

**If yes,**

**Number of pregnancies _____**

what have been the outcomes of your pregnancies:

Vaginal delivery _______

Forceps/vacuum delivery _______

C-section _______

Miscarriage _______

Ectopic/Termination _______

Please provide detailed information:

Have you had an attempt of vaginal delivery?

what have been the outcomes of this attempt (i.e. Cesarean Section, Normal Vaginal Delivery, Termination and or Miscarriage)?

Have you been advised to have a C section since your pelvic injury? YES NO

**If Yes** By whom you were recommended to do the C section and why?

1. Have you had the metal work inserted in your pelvis removed after the injury: **YES No**

If yes how long after the injury was removed (years/months):

1. Have you desired to be pregnant *since your pelvic injury*? **YES NO**
2. Are you afraid to become pregnant *since your pelvic injury*? **YES NO**
3. Have you been advised not to become pregnant *since your pelvic injury*? **YES NO**

**If yes**, By whom you were advised not to become pregnant and why?

Additional information:
